# Rhetorical Appeals and Tactics in New York Times Comments About Vaccines: Qualitative Analysis

**DOI:** 10.2196/19504

**Published:** 2020-12-04

**Authors:** John Gallagher, Heidi Y Lawrence

**Affiliations:** 1 Department of English, University of Illinois, Urbana-Champaign Urbana, IL United States; 2 Department of English, George Mason University Fairfax, VA United States

**Keywords:** vaccination, qualitative research, quantitative research, rhetoric, online comments, anti-vaccination, pro-vaccination

## Abstract

**Background:**

Improving persuasion in response to vaccine skepticism is a long-standing problem. Elective nonvaccination emerging from skepticism about vaccine safety and efficacy jeopardizes herd immunity, exposing those who are most vulnerable to the risk of serious diseases.

**Objective:**

This article analyzes vaccine sentiments in the New York Times as a way of improving understanding of why existing persuasive approaches may be ineffective and offers insight into how existing methods might be improved. We categorize pro-vaccine and anti-vaccine arguments, offering an in-depth analysis of pro-vaccine appeals and tactics in particular to enhance current understanding of arguments that support vaccines.

**Methods:**

Qualitative thematic analyses were used to analyze themes in rhetorical appeals across 808 vaccine-specific comments. Pro-vaccine and anti-vaccine comments were categorized to provide a broad analysis of the overall context of vaccine comments across viewpoints, with in-depth rhetorical analysis of pro-vaccine comments to address current gaps in understanding of pro-vaccine arguments in particular.

**Results:**

Appeals across 808 anti-vaccine and pro-vaccine comments were similar, though these appeals diverged in tactics and conclusions. Anti-vaccine arguments were more heterogeneous, deploying a wide range of arguments against vaccines. Additional analysis of pro-vaccine comments reveals that these comments use rhetorical strategies that could be counterproductive to producing persuasion. Pro-vaccine comments more frequently used tactics such as ad hominem arguments levied at those who refuse vaccines or used appeals to science to correct beliefs in vaccine skepticism, both of which can be ineffective when attempting to persuade a skeptical audience.

**Conclusions:**

Further study of pro-vaccine argumentation appeals and tactics could illuminate how persuasiveness could be improved in online forums.

## Introduction

### Background

Improving persuasive techniques when communicating with the public about vaccines is a long-standing concern. Although vaccination rates across the United States remain high, pockets of elective nonvaccination remain, which facilitate dangerous outbreaks [1,2]. Although newer movements that expand mandates appear to be successful in some locales [3], there is a continued need for persuasion in communities that resist such mandates and in cases where mandates are less viable, such as in the case of adult vaccinations.

This article asks specific questions related to online communication and vaccines: How do commenters, as readers of online newspaper articles, argue about vaccines? How might those arguments be better attuned to opportunities for persuasion? Finally, how can we better understand pro-vaccine arguments within a subset of both supportive and skeptical comments about vaccines? The study presented here analyzes 808 vaccine-specific comments posted on the *New York Times* (NYT) website’s online comment section. This analysis reveals two primary findings: (1) Both pro-vaccine and anti-vaccine comments rely on a thematically similar range of tactics, including appeals to children, science, and risks, though often drawing opposing conclusions. (2) Pro-vaccine comments rely on a set of appeals that offer uneven opportunities for audiences to be engaged and persuaded by pro-vaccine arguments.

As a space where people have active and agonistic arguments, online newspaper comment sections offer important insight into the persuasive contexts of vaccination discourses in real-world settings. Developing knowledge about persuasive tactics online can help researchers develop categories for persuasive appeals that users deploy when they discuss vaccines from pro-vaccine and anti-vaccine perspectives. In this study, vaccine comments were categorized according to pro-vaccine or anti-vaccine sentiments expressed, with common strategies and tactics used across comments identified. Critically, this work also addresses an important gap in online health communication by further documenting and analyzing the rhetorical appeals and tactics of *pro-vaccination* argumentation. While pro-vaccine websites [4], anti-vaccine websites [5,6], and anti-vaccination discussion forums [7-9] have been studied, to date no extensive studies have been conducted about the rhetorical appeals and argumentation strategies of pro-vaccination comments within a context of both pro-vaccine and anti-vaccine comments.

### Literature Review

What is known about vaccination sentiments and how they are communicated across a variety of spaces, including on the internet, is largely confined to understanding those who express skepticism about vaccines (eg, anti-vaxxers, vaccine refusers, or elective nonvaccinators). Existing studies locate sources of skepticism about vaccines in a broad range of concerns; the well-known yet refuted concern about the measles-mumps-rubella (MMR) vaccine and autism, “toxic overload” caused by vaccination, appeals to “natural” immunity and forms of disease protection, and personal choice/freedom are some of the primary reasons that parents refuse vaccines [8,10-17]. Other bodies of literature focus on the reasons for low uptake of flu vaccine and the rationales that adult patients have for not accepting the vaccine, including perceived constraints, concerns about a range of side effects, and lack of efficacy [18-20].

Still more articles focus on the deficits of those who refuse or question vaccines, examining “reasoning flaws” associated with vaccine concern [21], arguing for the ethical grounds for mandating vaccines [22,23], and counseling physicians and other healthcare providers on how to respond to vaccine concerns [24,25]. These studies frequently use analyses of hesitancy as a basis for these findings. These studies exist alongside popular press publications articulating the scientific and ethical problems associated with vaccine refusal or an “anti-vaccine movement” [26-28]. Another body of literature has studied specifically how these “anti-vaccination” beliefs are articulated online: typologizing skeptical sentiments that are popularly expressed online [5,6]; examining how risks are weighed and articulated in online chat forums [29]; and looking at ways skeptics use differing media platforms to spread anti-vaccination beliefs [7,30]. These studies offer a broad sense of the arguments and beliefs that parents present as rationales for not vaccinating or at least harboring concerns and skepticism about vaccination.

Newer research in this area has addressed differing tactics for online engagement [4-6,31-33], thereby widening the scope of analysis to both “pro” and “anti” sides of the issue on social media platforms. These studies demonstrate how online communication affords researchers new opportunities for: understanding how people communicate about vaccines; understanding a wider range of vaccine sentiments (outside of negative or skeptical ones only); and identifying new opportunities for persuasion or education about vaccines in online sites for public communication and interaction.

The work reported here contributes to this body of research by examining vaccine sentiments in online spaces, first categorizing pro-vaccine and anti-vaccine arguments and then discussing the ways in which their appeals and tactics interact to offer an understanding of the argumentative context in comments. This research also adds in-depth analyses of appeals and tactics in support of vaccines. Such understanding is important for gaining a broader sense of what the public thinks about the issue of vaccines in formats of online newspaper comments and how persuasive discourse works in online spaces. This article offers possible alternate sources of persuasion when those who support vaccines interact with those who are skeptical.

## Methods

### Design

The NYT was chosen for its established presence in the United States as a space of debate (it is a “paper of record”). The newspaper has a fourteen-person moderating team as well as machine learning technology from Google called Jigsaw. These tools work to keep comments relatively civil insofar as they filter out explicit language, egregious name-calling, and solicitation posts. We hypothesized that commenters from this newspaper would have a pro-vaccine inclination, a hypothesis that was borne out in our qualitative coding (see Qualitative Analysis Methods section).

In order to develop a targeted data set, we used a larger data set of comments (445,441) made on the NYT from May 1, 2015, to August 31, 2015. Comments were web-scraped using the newspaper’s application programming interface directions [34]. Comments were collected during the month of September 2015. Our rationale for this time period was to collect all comments for 4 months, which we believed to be a large enough sample to be representative of larger trends in commenting behavior. We then searched for the words “vaccine” and “vaxx” using a wildcard operator: vaccin*, vaxx*, vax*. This process yielded a final data set of 1101 comments about vaccines from the original 445,441 comments gathered. We have placed these comments into a publicly accessible database [35]. In 2 cases, a pro-vaccine comment was repeated, but as the repeated comments were on different threads, we included them in the data set. To develop a deeper analysis of this data, we conducted a qualitative analysis on the data. We note that these comments, whether pro-vaccine or anti-vaccine, are generally civil, meaning they avoid name-calling and offensive language and are articulated in ways that do not employ heated exchange (capitalizations, use of multiple exclamation points, etc). This civility is likely a product of the NYT’s moderation of the forum but could also be indicative of civility concerning this topic in this space during this timeframe.

### Qualitative Analysis Methods

We coded the comments as pro-vaccine, anti-vaccine, or not applicable. To classify anti-vaccine comments, we decided comments needed to be against vaccines or demonstrate some skepticism toward them. To classify pro-vaccine comments, we looked for comments that advocated the use of vaccines, broadly conceived. The two authors of this paper initially coded the first 100 comments separately and then met to discuss whether we thought this was a productive schema. We confirmed that we were in general agreement about the schema and then proceeded to code the remaining comments. All coding was done separately. We then compared the coding. We disagreed on 21.80% (240/1101) of comments. We did not code these comments thematically due to rater disagreement. The remainder of the comments, which had been agreed upon, broke down as follows: 602 (54.68%) were pro-vaccine, 206 (18.71%) were anti-vaccine, and 53 (4.81%) were neither pro-vaccine nor anti-vaccine and were not coded thematically.

This process yielded 808 comments that we categorized thematically. After the previous categorization schema, we then coded for themes, appeals, and tactics, drawing on a version of guided grounded theory and specifically looking for rhetorical appeals or arguments that speakers used in their posts. We separately coded the initial 100 comments according to appeals and argumentation. Comments could be coded multiple times to account for multiple appeals. We then met to discuss this coding. We eventually settled on three primary appeals as emerging from the data: *dissoi logoi*, appeals to science, and appeals to the “public good”; these appeals were shared by pro-vaccine and anti-vaccine comments yet were obviously used to achieve different ends or conclusions about vaccines. We subsequently proceeded to thematically code the remaining data set, assigning individual themes for the authors to code. We also made notes of less frequent appeals and tactics as they emerged in the data. Within this context of understanding the appeals and tactics used across vaccine comments, we pursued additional, in-depth analysis of pro-vaccine comments because of their novel abundance in this data set.

## Results

Below are the pro-vaccine (Textbox 1) and anti-vaccine (Textbox 2) appeals and tactics. The numbers in parentheses are the numbers of comments expressing each appeal or tactic. The appeals and tactics were not mutually exclusive, and many comments contained multiple appeals and tactics. We do not list appeals or tactics that occurred fewer than 5 times.

**Appeals and tactics of pro-vaccine comments (n=602).**

**Appeals**

- The public good (234)
- Science and expertise advocacy (166)
- *Dissoi logoi* (constructing opposing viewpoints) (102)
- Personal experience or personal ethos (79)
- The social good (68)
- Accepting small risks (50)
- Attributing vaccine denial to the other political spectrum (31)

**Tactics**

- Direct (use of username or name) or indirect (use of second person) address of vaccine refusers (129)
- Ad hominem (name-calling) (79)
- Debunking autism-vaccine connections (48)
- Referring to evidence (use of hyperlink, typically to government websites) (39)
- Asking anti-vaccine proponents to conduct research (17)
- Debunking mercury-vaccine connections (6)
- Debunking thimerosal connection (6)

**Appeals and tactics of anti-vaccine comments (n=206).**

**Appeals**

- Skepticism: toward institutions (56); toward science (22); toward vaccination rates (14); toward herd immunity (8)
- Concern over money in “big pharma” or “big money” (the money in the pharmacy industry; used either phrase) (33)
- Risk to pregnant individuals (31)
- Personal freedom (29)
- Concern over number of vaccines administered (28)
- Appealing to “complex issues” (some form of the word “complex” without a specific issue mentioned) (22)
- Concern over thimerosal, mercury, and additives in vaccines (17)
- Criticism that vaccines are often for preventable diseases (14)
- Concern over thimerosal in general (12)
- There needs to be more market competition for testing and developing vaccines (11)
- Attempts to connect vaccines to non-autism diseases (7)
- Risk is logical (6)
- Children at risk of vaccines (5)

**Tactics**

- Claiming not to be 100% against vaccines (31)
- Referring to evidence (use of hyperlink, typically to commercial websites) (20)
- Scientific terminology used incorrectly (17)
- Direct or indirect address to pro-vaccine commenter (use of username or second person) (16)
- Asking other commenters to conduct research (11)
- Against ad hominem (8)
- Ad hominem (7)
- Criticism of “other side” (7)
- Direct address to author of article (use of author’s first name, last name, or both) (5)

## Discussion

Qualitative analyses demonstrate that there were common appeals and tactics used across pro-vaccine and anti-vaccine discourses, but also some notable differences in the arguments and argumentative patterns of different positions. This analysis focuses in particular on a number of trends in pro-vaccine argumentation that make opportunities for persuasion problematic and that, if addressed, could help improve the persuasive quality of pro-vaccine discourse online.

### Overview

Pro-vaccine comments drew on a narrower range of appeals, tactics, and themes whereas anti-vaccine comments had a broader range of appeals, tactics, and themes. We attribute this difference to at least two causes. First, our binary coding schema of pro-vaccine and anti-vaccine led us to categorize many comments that expressed skepticism over some vaccines or approaches to vaccination as “anti-vaccine.” Comments that were vehemently, completely anti-vaccine were lumped into the same category as comments that were pro-vaccine for childhood vaccinations (eg, MMR, chicken pox) while being anti-vaccine for newer vaccinations (eg, human papillomavirus [HPV]). Moreover, we found many comments that expressed hesitation over vaccination scheduling. Second, being pro-vaccine involves advocating *for something.* Conversely, anti-vaccine comments simply need to cast skepticism on that something. Therefore, the wider set of appeals, tactics, and themes used by anti-vaccination comments makes sense: skepticism involves creating some level of doubt, even if minor. Anti-vaccine comments thus need only discuss a concerning issue, no matter how small or insignificant it is.

In broad strokes, anti-vaccine comment strategies are more diverse and heterogeneous than pro-vaccine comment strategies. Anti-vaccine commenters appealed to science’s fallibility, the minute presence of risk, and freedom of choice. Anti-vaccine commenters appealed to the debunked research conducted by Andrew Wakefield and others. Anti-vaccine commenters used hyperbole frequently, notably in increasing the number and frequency of vaccine shots. The concept of risk is particularly illustrative. On the one hand, pro-vaccine comments discuss risk in a relatively homogeneous way: it is something to be minimized, and comments recognize the low level of risk. On the other hand, anti-vaccine comments were concerned over realistic types of risk *and* radically untrue types of risk, including overstatement and hyperbole, which was dramatically uneven in the data set. Some anti-vaccine commenters argued that physicians wanted to give babies dozens of vaccines and argued that all vaccines had high risks. Other commenters expressed less hyperbolic concerns, such as worries about spacing of the MMR vaccine and a desire to discuss risk with a practitioner before accepting a vaccine.

### Pro-Vaccine Comments

We noted early in our analysis that the data set included a novel quantity of pro-vaccine comments. We suspect this number of pro-vaccine comments is a product of the NYT’s typical readership as well as the effect of comment moderation. However, as noted in the literature review above, with the typical focus on anti-vaccine comments and deficit approaches to correcting this stance, less information is established in the literature on the argumentative patterns of pro-vaccine advocates. We report on these trends below to add novel findings to the literature, but also to point to spaces where pro-vaccine advocates could improve the persuasiveness of their commentary.

Overall, we found that pro-vaccine comments included three dominant appeals and tactics. The first category was appeals to science and expertise. Science and expertise advocacy includes appeals to science as a black box (eg, advocating for trusting “the science”, as in the words themselves). These appeals also include trusting scientists and experts in general (most frequently the “medical community”) as well as specifically by a proper name. These appeals included advocating for rationality and logic and attacking anti-science viewpoints (eg, “science-deniers”, lack of “scientific links”, “anti-science ignorance”). Less frequently, commenters in this category mentioned scientific concepts such as controlled studies, falsifiability, and hypothesis testing. Comments that used this appeal were heavily anti-anecdote and requested evidence. They corrected scientific inaccuracies (such as the number of required vaccines, side effects of vaccines, and use of mercury). We note that this operates in opposition to skeptical comments about trusting individual forms of knowledge, such as personal experience. This creates a clash between these two perspectives, wherein the concept of science becomes a stand-in for trustworthy forms of expertise, and skeptical perspectives denounce or diminish that perspective through—often incorrect—critiques of science. Although such corrective forms of communication might seem like a helpful, even persuasive, intervention, previous studies of vaccine sentiment have indicated that such measures can have a “backfire effect,” causing people to more firmly believe incorrect beliefs upon having them corrected [36].

Second, for vaccine proponents, vaccination is associated with the common good or what is generally perceived to be good for the public. A remarkable number of pro-vaccine comments focused on appealing to the public and social good of vaccines. *Public good* took multiple forms, including herd immunity, the safety of children and older adults, and the sickness and deaths of young children from vaccine-preventable diseases. These comments often mentioned legal liability for those who do not vaccinate; the extension of this logic implies a prevention of epidemics and increase in herd immunity. Comments that appealed to the public good of vaccines frequently made other non–vaccine-related claims about the public good, including climate change and economic equality.

Comments that focused on what we label the *social good*, while related to the public good, made use of historical statistics and information to discuss the quality of life that vaccines brought about. We note that this appeal is often used in conjunction with personal experience and *ethos*. Commenters frequently recall their childhoods, often detailing the suffering of other children from vaccine-preventable diseases. They do so in order to discuss the social good (and social progress) that vaccines have society. These appeals, though they offer the potential for persuasion through their use of anecdote and narrative—devices that can be more persuasive for a skeptical public—also operate in direct opposition to skeptical appeals to individualism rather than collective good. Thus, the ideological gap between the “good story” these conflicting narratives tell (one of protecting individual rights versus achieving collective good) lessens their potential to operate persuasively for skeptical readers.

Third, vaccine proponents construct opponents’ arguments as a way of establishing their position and amplifying their support of vaccines, an appeal called *dissoi logoi*, or construction of oppositional or contrasting arguments [37]. *Dissoi logoi* in classical argumentation originated as a mechanism for understanding and examining opposing sides. In its best form, *dissoi logoi* allows the speaker to see and articulate an issue from someone else’s perspective, but it can lead to specious arguments as well when oppositional arguments are misunderstood, weakly constructed, or incorporate their own fallacies. When pro-vaccine arguments employed *dissoi logoi*, they used it to approximate or describe vaccine skepticism from within their own position of support, leading to reductive and ad hominem attacks associated with their estimation of the “opposing side.” These appeals are particularly potent sources of creating opposition, rather than opportunities for persuasion, since restated arguments frequently create opposing arguments not worthy of refutation.

For example, the following comment conflates concern about HPV vaccine with laziness or ignorance:

[Rick Perry, Governor of Texas who endorsed an unpopular mandate for HPV vaccine,] should have gone through with it for their own good. This vaccine, if taken early enough, will prevent cancer. The people in Texas who are opposed are a bunch of religious bozos who think that getting this vaccine will make it more likely that their children will have premarital sex. That is sheer ignorance.

This comment engages in *dissoi logoi* through constructing the argument of vaccine skeptics, invoking “the people in Texas who are opposed” to the policy. This comment features multiple ad hominem tactics insulting anti-vaccine perspectives or anti-vaccine commenters, including calling skeptics “bozos” and citing them as a danger to efforts to prevent cancer. Ad hominem was significantly more prevalent in pro-vaccine comments than in anti-vaccine comments and is classically problematic as a persuasive act, since personal attacks can cause defensiveness, thus diminishing persuasive appeal.

Finally, it is worth noting that there also appear to be ideological components to both the pro-vaccine and anti-vaccine comments. The ideology that undergirds pro-vaccine comments includes concern for public welfare and society as a functioning whole, whereas the anti-vaccine comments have an ideology of concern for individual welfare and autonomy. From our reading of the comments, these ideologies operate independently of typical US politics (conservative vs liberal) because many comments attribute the anti-vaccine perspective to the “other side.” Often, if a comment appears to have a traditionally conservative leaning, then it will attribute the anti-vaccine movement to liberals, noting that liberal places in California or Oregon have outbreaks of preventable diseases. Anti-vaccine perspectives were also attributed by pro-vaccine commenters to people who are anti-science, anti–genetically modified organisms (GMOs), and anti–nuclear power, and climate change deniers. For instance, one emblematic comment reads as follows:

I find it very hard to respect the resistance to empiricism common to the anti-GMO crowd, anti-vaccine fanatics and climate change deniers. The widespread popular refusal to come to conclusions based on evidence suggests a dim future for us all. We will need science, we will need technology, and GMOs will have to be one of the tools available to us if we hope to feed the nine billion people expected to share a warming and highly stressed planet at mid-century.

On the other hand, if a comment appears to have a liberal leaning, it will attribute the anti-vaccine movement to conservatives. These comments often noted that the concept of “personal choice” undermines herd immunity and the health of the public. As an emblematic example, one commenter writes the following:

Well, in large part because of the policies--family planning, vaccination, the Peace Corps, the green semi-revolution and so on--that the Right foight [sic] tooth and nail against spending any money on? By the way: that progress, and in many ways it IS [sic] progress, did come at a price, you know. Take a look at the bill, which includes warmong [sic] up the planet and polluting a fair old chunk of it.

We make this observation as a way of noting that, although varying ideologies are operating within each perspective, a clear political alignment cannot be discerned.

### Limitations and Future Research

While there are several limitations of this study, we first address a strength of this study: the agreement between the two authors. We had not worked together before but came to surprising agreement (861/1101 comments, 78.20%) about whether to categorize comments as pro-vaccine or anti-vaccine (or not applicable). Following this degree of agreement, we believe that most vaccine-related comments in our data set ascribe to either advocacy of vaccines or skepticism of vaccines.

We urge caution with our findings due to the limited size of the data set (808 comments), the venue in which the comments were made (the NYT), and our own coding schema that did not analyze comments that we disagreed upon or comments that we categorized as neither pro-vaccine nor anti-vaccine. The greater number of pro-vaccine comments may be the result of the screening process by the moderating team at the NYT, the Jigsaw machine learning technology, or both. More conceptually, however, our method has not addressed the many other issues present in these comments, including US politics, local discussions, reader-to-reader relationships, and so forth. In this sense, we have focused our analysis to a particular theme at the detriment of examining how those themes intersect with other rhetorical moves made.

A more conceptual limitation is that, because the data we used were public comments, the commenters may not see themselves as attempting to be truly rhetorical (eg, persuasive). In this sense, these findings have qualifications, the most relevant being that the appeals and tactics are not necessarily geared at being highly persuasive. Nevertheless, the detail offered in these comments adds information to the online conversation around vaccination, notably the finding that the pro-vaccine and anti-vaccine comments deploy several similar strategies while providing foils to one another.

A major challenge about this project has to do with the heterogeneous debates under the broad term of vaccine. Within our data set, vaccines were discussed, many of which were childhood vaccinations (MMR, chickenpox, etc). However, other types of vaccines were discussed, such as the Ebola vaccine (the NYT ran an article about this in 2015). Further, vaccines were mentioned in tandem topics that were not directly related (for instance, vaccines were frequently discussed alongside genetically modified food topics). In our methodology, we have flattened the discussion focus in order to analyze the appeals and tactics surrounding vaccine debate.

As a result of these limitations, we have several suggestions for future research. These include increasing the number of comments analyzed, examining other venues, and running more advanced computer analysis (corpus linguistics and natural language processing). Analyzing vaccine-related comments outside the binary of pro-vaccine and anti-vaccine may be useful for future research. In this article, we have also not analyzed the comments about which we disagreed in our coding schema or the comments that we agreed were neither pro-vaccine nor anti-vaccine. Analyzing these types of comments may reveal additional rhetorical appeals or analytical tactics as well as nuanced relationships between the binary of pro-vaccine and anti-vaccine perspectives. Finally, because comments are an informal place where persuasion might happen, researchers could insert different comments that deploy varying levels of persuasive appeals in order to determine which appeals work more effectively than others. Research could then document reactions to these comments as well as survey and interview commenters to evaluate the effectiveness of specific appeals.

### Conclusion

When it comes to vaccination, the stakes are high. With the urgent public health need to achieve high rates of universal vaccination, each encounter with a person about vaccines can be an opportunity to strengthen public trust in and acceptance of vaccines. We offer the previous analyses as a way for pro-vaccine commenters across public and professional spaces to consider how their tactics can be more persuasive to skeptics. Simply dismissing skeptics will not change their minds, and appealing to vaccine supporters does not actively engage with changing the status quo. Our analyses of the pro-vaccine comments may help to guide future studies attempting to identify how and why vaccine skeptics are persuaded in online, participatory environments.

## Abbreviations

GMO: genetically modified organism
HPV: human papillomavirus
MMR: measles-mumps-rubella
NYT: New York Times
